# Biochemical Characterization and Application of a Detergent Stable, Antimicrobial and Antibiofilm Potential Protease from *Bacillus siamensis*

**DOI:** 10.3390/ijms24065774

**Published:** 2023-03-17

**Authors:** Hasan Tarek, Kyung Bin Nam, Young Kyun Kim, Suzia Aktar Suchi, Jin Cheol Yoo

**Affiliations:** Department of Pharmacy, College of Pharmacy, Chosun University, Gwangju 501-759, Republic of Korea

**Keywords:** biochemical characterization, antimicrobial protease, antibiofilm, *Bacillus*

## Abstract

Proteases are important enzymes that are engaged in a variety of essential physiological functions and have a significant possible use in industrial applications. In this work, we reported the purification and biochemical characterization of a detergent stable, antimicrobial, and antibiofilm potential protease (SH21) produced by *Bacillus siamensis* CSB55 isolated from Korean fermented vegetable kimchi. SH21 was purified to obtain homogeneity via ammonium sulfate precipitation (40–80%), Sepharose CL-6B, and Sephadex G-75 column. By analyzing the SDS-PAGE and zymogram, it was determined that the molecular weight was around 25 kDa. The enzyme activity was almost completely inhibited in the presence of PMSF and DFP, which indicated that it was a member of the serine protease family. SH21 showed excellent activity with a broad range of pH and temperature, with its maximum pH of 9.0 and temperature of 55 °C. The enzyme had estimated K_m_ and V_max_ values of 0.197 mg/mL and 1.22 × 10^3^ U/mg, respectively. In addition, it preserved good activity in the presence of different organic solvents, surfactants, and other reagents. This enzyme showed good antimicrobial activity that was evaluated by MIC against several pathogenic bacteria. Furthermore, it exhibited strong antibiofilm activity as determined by MBIC and MBEC assay and degraded the biofilms, which were analyzed by confocal microscopic study. These properties established that SH21 is a potent alkaline protease that can be used in industrial and therapeutic applications.

## 1. Introduction

There are many different types of industrial enzymes on the market. However, proteases represent one of the most valuable classes, containing around 40% of the industrial enzyme market share worldwide [1]. In a few industries, they are employed for a variety of uses, including laundry detergent, foodstuff, pharmaceuticals, rawhide, peptide synthesis, and silver recovery from used X-ray films [2]. The most frequently used commercial enzymes are microbial proteases, particularly those from *Bacillus* sp., which find widespread use in detergent composition and other industrial uses. Applications in bioengineering and biotechnology are interested in proteases with a higher activity and stability in higher alkaline ranges, because laundry detergents typically have a pH between 9.0 and 12.0, where they are mostly used. The pH of the cleansing solution, washing temperature, and detergent composition are only a few of the variables that affect how well detergent proteases work. Consequently, proteolytic enzymes used in detergent compositions need to have a substantial amount of activity.

Numerous investigations have been carried out on alkaline proteases from strains that produce high yields, but bleach-stable enzymes are rarely found, excluding limited studies [3,4]. Furthermore, several alkali proteases isolated from various *bacilli* strains have been purified and characterized [5,6]. Due to their broad specificity, a few commercial proteases, such as subtilisin isolated from *Bacillus* sp., have been chosen for detergent formulation [7,8]. This enzyme displayed its maximum activity at pH levels of 8.0 to 10.0. Nowadays, researchers are looking into new sources of proteases due to the rising demand for proteases with specific characteristics. Unlike those isolated from plants and animals, microbial proteases often exist extracellularly and are directly released by the producer into fermentation media that ease the subsequent processing, purification, and enzyme recovery.

The opportunity for proteases, not only as proteolytic enzymes but also as antimicrobial agents for many diseases and infections, could have an enormous impact on medical and various clinical treatments. Protease enzymes are produced by several microorganisms and are implicated in metabolic regulation and the development of numerous infectious maladies. Recently, antimicrobial resistance has been a significant health concern worldwide, leading to ten million deaths yearly as treatment failure increases due to the ineffective use of antibiotics [9]. The proteases from *Bacillus subtilis*, *Bacillus* sp., and *Artocarpus heterophyllus* have been reported to be antimicrobial in nature against diverse pathogenic bacteria [10,11,12].

Microbial protease has also been reported to inhibit biofilm formation due to its anti-biofilm properties. It is considered an eco-friendly and cost-effective method because of its non-toxic and biodegradable nature. Biofilms are surface-attached bacterial cells enclosed within an extracellular matrix and arranged in complex communal tertiary structures. Bacteria produce and secrete proteins, lipids, polysaccharides, and nucleic acids, called extracellular polymeric substances (EPS), which make up approximately 95% of the biofilm matrix. Some opportunistic bacteria, such as *E. coli*, *Staphylococcus*, *Pseudomonas*, etc., form biofilms that impair healing in chronic and acute dermal wounds and result in reinfections and sepsis [13,14]. Therefore, it is crucial to treat infectious diseases to prevent biofilm formation and disrupt existing biofilms. Some heavy metals, including silver, mercury, copper, nickel, zinc, and other chlorinated compounds, have been used for the dispersion of biofilms. However, these chemicals are no longer used because of their severe health risk [15]. It is necessary to replace chemicals with enzymes to remove biofilms due to the various limitations of such chemicals.

Antibiofilm potential enzymes such as protease, glycosidase, and DNases have the capacity to damage the extracellular matrix, releasing planktonic cells and their constituents that can be more simply accessed by antimicrobial agents [16,17]. Proteases are considered the best enzymes for removing biofilms since proteins are significant components of biofilms [18]. Various microorganisms produce proteases that degrade biofilms, such as proteinase K, aureolysin, spl protease, and staphopain A and B [17]. To the best of our knowledge until now, a detergent-stable protease that possesses both antimicrobial and antibiofilm activity, and is purified from the *Bacillus siamensis* strain isolated from kimchi has never been studied. This report aimed to explore the stability and compatibility with various commercial detergents of protease SH21 as a possible candidate for industrial applications, mainly in the detergent formulation and the medical areas.

## 2. Results and Discussion

### 2.1. Screening, Identification, and Phylogenetic Tree of Protease-Producing Strain

**Figure 1:**
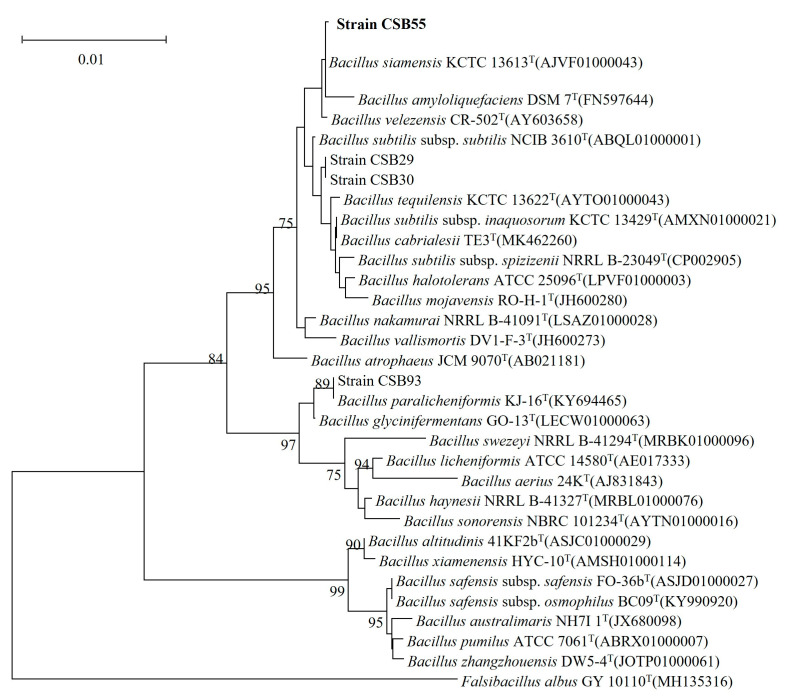
Neighbor-joining phylogenetic tree reconstructed based on 16s rRNA gene sequences displaying relationships between CSB55 and several nearly related taxa of the genus *Bacillus*.

In the present study, about eighty new bacterial strains were isolated from kimchi, and they were found as protease producers on casein agar media at pH 9 based on the clear area developed upon the hydrolysis of casein. The proportion of the clear area to colony diameter was used to choose strains with significant protease-producing capabilities. Among those isolates, 17 strains exhibited a clear hydrolytic zone on casein agar media after 24 h of incubation at 37 °C. Among them, a strain called CSB55 produced the highest clear hydrolytic zone and exhibited a high protease activity, thereby being chosen for further study. The identification of the strain CSB55 was made according to Bergey’s Manual of Systemic Bacteriology based on morphologic and biochemical properties. A phylogenic tree was then constructed from a 16S rRNA gene sequence that exhibited 99.99% similarity to *Bacillus siamensis* KCTC 13613 (Accession no: AJVF01000043) (Figure 1). This isolate has been assigned to *Bacillus siamensis* CSB55 based on the results of this study.

### 2.2. Enzyme Production and Purification of Protease SH21

**Figure 2:**
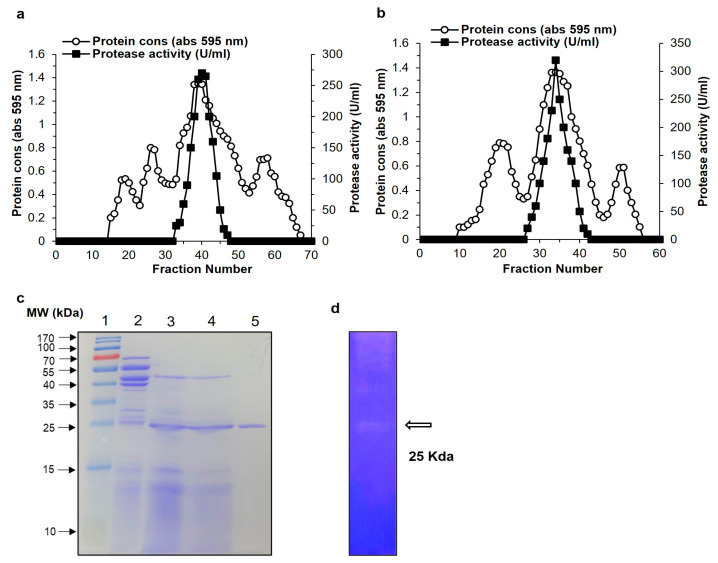
Purification of protease SH21 from *Bacillus siamensis* strain CSB55. Chromatography profile of SH21 on gel filtration using (**a**) Sepharose CL-6B (80 cm × 1.8 cm) and (**b**) Sephadex G-75 column (1.5 × 20 cm). (**c**) SDS-PAGE (12%) of purified protease SH21. Lane 1, protein molecular weight marker standard from 10 to 170 kDa. Lane 2, crude extract. Lane 3, ammonium sulfate precipitation (40–80%). Lane 4, sample after Sepharose CL-6B chromatography. Lane 5, purified protease SH21 after Sephadex G-75 column. (**d**) Zymography of purified SH21.

Protease production media was used to produce the protease enzyme from *Bacillus siamensis* CSB55, and casein was used as a protease inducer in the medium. Enzyme production began at 16 h and increased to its highest level at 64 h at 37 °C. The crude enzyme preparation of protease from *Bacillus siamensis* CSB55 was obtained using 1500 mL of culture supernatant centrifuged at 10,000 rpm for 30 min at 4 °C. The enzyme was purified by three-step procedures with ammonium sulfate precipitation (40–80%) and using two size exclusion chromatography including Sepharose CL-6B and Sephadex G-75. All steps of purification are summarized in Table 1. The elution outlines of Sepharose CL-6B and Sephadex G-75 are presented in Figure 2a,b. As a result of the final purification step, the enzyme SH21 had a yield of 16.23%, a specific activity of 2926.67 U/mg, and was 23.09-fold pure.

### 2.3. Molecular Weight Determination and N-terminal Amino Acid Sequence

SDS-PAGE was used to determine the molecular weight of protease SH21 from *Bacillus siamensis* CSB55. The purified protease exhibited a single band on the SDS-PAGE, suggesting its homogeneity and it showed molecular weight of about 25 kDa (Figure 2c). In comparison with other *bacillus proteases*, purified SH21 had a lower molecular weight than *Bacillus stearothermophilus* (28 kDa) [19], *Bacillus amyloliquefaciens* (43 kDa) [20], but higher compared with those produced by *Bacillus subtillis* (17.1 kDa) [21], and *Bacillus licheniformis* (19.7 kDa) [22]. For the purified sample, the zymogram activity specified a caseinolytic zone with a projected molecular weight of 25 kDa, which was seen co-migrating with a protein. (Figure 2d). These findings strongly recommend that SH21 is a monomeric protein, similar to those found in other *bacillus* proteases. The N-terminal sequences of purified SH21 were determined to be QTGGSFFEPFNSYNSGLWQKANGYS. The alignment analysis of the amino acid sequences of SH21 with other similar proteins from various *Bacillus* species are showed in Table 2.

### 2.4. Biochemical Characterization of Purified Protease SH21

#### 2.4.1. Effect of pH on the Activity and Stability of Protease SH21

**Figure 3:**
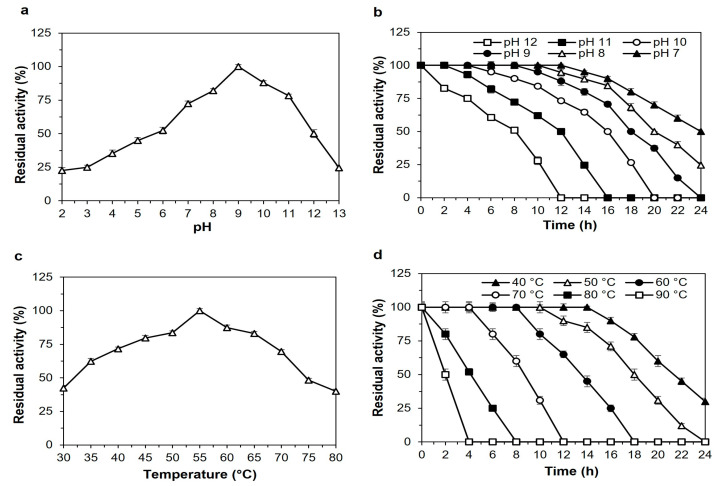
Effect of pH on the (**a**) activity and (**b**) stability of purified protease SH21 from *Bacillus siamensis* strain CSB55. To check the effect of pH on purified SH21, enzyme assay was carried out using different pH ranges (2–13.0) at 55 °C. The enzyme activity at pH 9.0 was taken as 100%. Effects of (**c**) thermoactivity and (**d**) thermostability on purified SH21. To determine thermoactivity, enzyme SH21 was incubated at different temperatures ranging from 30 to 80 °C with optimum pH (9.0), while thermostability was evaluated at various temperatures ranging from 40 to 90 °C for 24 h at 2 h intervals. Non-heated enzyme activity was considered 100%. Each result presents the mean of three separate experiments (±) standard deviation.

*Bacillus* sp. proteases are well-known for their ability to tolerate acidic, alkaline conditions [23]. These are suitable criteria for industrial uses of protease enzymes. The enzyme SH21 exhibited activity over a wide range of pH (3.0–13.0) and was highest at pH 9.0 (Figure 3a). The residual activities were 52% and 50% at pH 6 and 12, respectively. However, protease SH21 displayed excellent activity from pH 7 to 11, which was around 72 to 100%. The stability of protease was also good at a large variety of pHs from 7 to 12 (Figure 3b). The half-life of enzyme SH21 at pH 7, 8, 9, 10, 11, and 12 was 24, 20, 18, 16, 12, and 8 h, sequentially. SH21 is more effective at an alkaline pH in comparison to commercially available detergent enzymes such as Alcalase (Novozymes BiopharmaDK A/S, Bagsvaerd, Denmark), which is produced by *Bacillus licheniformis* and has a maximum activity range of 8 to 9 [24]. The high activity and stability displayed by SH21 at alkaline conditions is an incredibly crucial property given the potential strength of future industrial applications, especially in detergent preparations, since they are mostly formulated at pH 7–11 [25]. SH21 protease exhibits higher pH stability than currently commercialized detergent proteases on the market.

#### 2.4.2. Effect of Temperature on the Activity and Stability of Protease SH21

Thermostability is a crucial factor when determining the potential of proteases for use in an industrial process that demands high temperatures. Usually, thermostable proteases are used in industrial processes that are not possible at room temperature. The protease activity was remarked at pH 9.0 over a broad range of temperatures from 30 to 80 °C with a maximum of 55 °C (Figure 3c), which is higher than those of protease enzymes *Bacillus pumilus* (40 °C), *Bacillus subtilis* (50 °C) [26], and lower than those of *Bacillus subtilis* (60 °C) [21], and *Bacillus* sp. (70 °C) [27]. The thermostability profile of SH21 is presented in Figure 3d. Protease SH21 showed good activity (≥80%) after being incubated at 40, 50, 60, 70, and 80 °C for 16, 14, 10, 6, and 4 h, respectively. The enzyme activity remained around 50% when it was incubated at 80 and 90 °C for 4 and 2 h, separately. So, this attribute indicated that protease SH21 had excellent thermal stability. Higher temperature is critical in chemical reactions because of the more excellent solubility of substrates, superior mixing, quicker reactions, decreased stickiness, and reduced possibility of bacterial contamination [28].

#### 2.4.3. Effect of Inhibitors, Metal Ions, Surfactants, and Bleaches on Protease Stability 

Enzyme inhibitors are elements that interact with enzymes (temporary or permanent) to inhibit the rate of an enzyme-catalyzed reaction or prevent enzymes from functioning correctly. Proteases are categorized according to how sensitive they are to specific inhibitors [29]. The effects of different protease inhibitors and other chemicals are summarized in Table 3. The inhibition of protease activity by PMSF and DFP led to the conclusion that the co-produced protease belongs to the serine protease family. PMSF and DFP act as covalent modifiers of the enzyme’s active site, inhibiting each serine protease [30]. According to the literature, serine proteases account for nearly one-third of all proteases [31]. Other inhibitors, such as TPCK, TLCK (chymotrypsin alkylating agent), SBTI (soybean trypsin inhibitor), and benzamidine (trypsin competitive agent) did not exhibit any inhibitory effect on the protease activity. Furthermore, a thiol reagent (2-mercaptoethanol, iodoacetamide, DTNB, leupeptin) and pepstatin A (aspartyl protease inhibitor) had almost no substantial impact on the SH21 activity. EDTA (10 mM) and EGTA (1 mM), both metalloprotease chelating inhibitors, had reduced SH21 activity to 87% and 85%, respectively, when they were added to purified protease, where they may serve as cofactors. Chelators’ insensitivity to the enzyme would be a desirable attribute because they used detergent compositions as water softeners and stain removers [1].

The effect of numerous metal ions and other chemicals on the SH21 activity was also examined. So, the SH21 activity was lifted in a remarkable manner by 135%, 120%, 125%, and 145% following the addition of Mg^2+^, Mn^2+^, Zn^2+^, and Fe^2+^ separately, at a final concentration of 3 mM. This result suggested that the enzyme required magnesium, manganese, zinc, and iron to function optimally. The enzyme activity was unchanged by Ca^2+^, Na^+^, and Cu^2+^ and was almost entirely prevented by Ni^2+^, Co^2+^, Cd^2+^, and Hg^2+^ at the same concentration as other metallic ions. Increasing the activity of protease with Mn^2+^ and Mg^2+^ showed that the metallic ions had a defensive attitude against denaturation, therefore allowing the enzyme to retain its activity at higher temperatures [2,32]. The poisonous metallic ions caused the denaturation of proteins, including enzymes, by attaching to a series of chemical ligands. Heavy metallic ions have an inhibiting effect that has been widely documented in the literature [33]. In addition, SH21 was highly stable with the addition of several anionic and non-ionic surfactants such as SDS, Tween 20, Tween 80, and Triton X-100 at 1% concentrations, and the enzyme retained its activity around 85%, 90%, 88%, and 92% accordingly. The enzyme stability was also investigated by incubating it with H_2_O_2_ as well as NaClO (1% *v*/*v*) as bleaching agents, and it maintained 80% and 83% activity, respectively. However, there are limited studies on alkaline proteases in the presence of surfactants and bleaching agents [34]. In general, proteases tend to be stable in various detergent components, but they are unstable in bleaching agents. Protease SH21 showed significant stability and activity with surfactants, bleaching agents, and other chemicals, which are crucial properties for use in detergent formulations and other industrial applications.

#### 2.4.4. Effect of Organic Solvents on the Activity and Stability of Protease SH21

**Figure 4:**
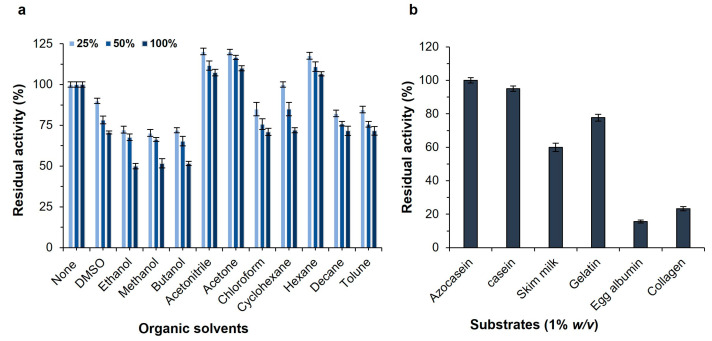
(**a**) Effect of organic solvents on the activity of purified protease SH21 from *Bacillus siamensis* CSB55. (**b**) Effect of different substrates on purified protease SH21 from *Bacillus siamensis* CSB55. Vertical bars indicate the mean (n = 3) ± standard deviation.

The influence of several organic solvents was examined on the protease stability at concentrations of 25, 50, and 100% (*v*/*v*) for 24 h (Figure 4a). In most cases, interactions between an enzyme and a solvent substantially affect the secondary and tertiary structures of the enzyme, as well as its activity and stability. Surprisingly, the SH21 activity was marginally enhanced in the presence of acetonitrile, acetone, and hexane. There was evidence that the enzyme activity could be increased due to some water molecules that may have been replaced by solvent molecules, thereby changing the structure of the enzyme in the presence of solvents [23]. The enzyme preserved (70–100%) the original activity with the addition of dimethyl sulfoxide (DMSO), chloroform, cyclohexane, decane, and toluene at all concentrations. In addition, protease maintained its activity by more than 50% after it was added to the enzyme solution of 100% of ethanol, methanol, and butanol, while it showed over 65% activity with the same solvents at 25 and 50% concentrations. The enzyme SH21 was found to be significantly more stable in organic solvents compared to other microbial proteases from *Bacillus* sp. [35,36]. These results revealed that protease SH21 is highly stable in various organic solvents, which is vital for industrial purposes. The most useful application of a solvent-resistant protease is the synthesis of peptide and ester, which is done in non-aqueous environments in industries.

#### 2.4.5. Substrate Specificity and Enzyme Kinetics of Protease SH21 

The substrate specificity of alkaline proteases is generally wide, and they can hydrolyze a broad range of substrates. Various substrates were investigated to find out the best substrate for a proteolytic reaction including azocasein, casein, skimmed milk, gelatin, egg albumin, and collagen (Figure 4b). Azocasein was shown to be the most suitable substrate for the purified protease SH21, and the residual activity was 100%. Moreover, the enzyme was able to hydrolyze other substrates such as casein, skim milk, and gelatin with residual activities of 95%, 60%, and 77%, accordingly, but egg albumin and collagen showed poor hydrolysis. This result indicated that protease SH21 has a broad substrate specificity. The Michaelis–Menten constant (K_m_) and the maximum reaction velocity (V_max_) were established for the purified enzyme SH21 employing azocasein as the substrate. The K_m_ value of an enzyme suggests the affinity of the enzyme to its substrate. The minor K_m_ and higher V_max_ represent the greater affinity and catalytic efficiency of the enzyme for its substrates. The purified protease showed K_m_ and V_max_ values of 0.197 mg/mL and 1.22 × 10^3^ U/mg, respectively, and it was analyzed by a Lineweaver–Burk plot. Protease SH21 from *Bacillus siamensis* CSB55 had a lower K_m_ value than other proteases from *B. subtilis* (1.17 mg/mL) [37], *B. megaterium* (0.722 mg/mL) [38], and *B. licheniformis* (1.60 mg/mL) [39]. However, this result indicated that the enzyme SH21 showed a very high affinity to its substrate since its K_m_ value is much lower than other proteases obtained from different *Bacillus* species.

### 2.5. Application of Protease SH21 

#### 2.5.1. Commercial Detergent Stability and Stain Removal Ability of Protease SH21

**Figure 5:**
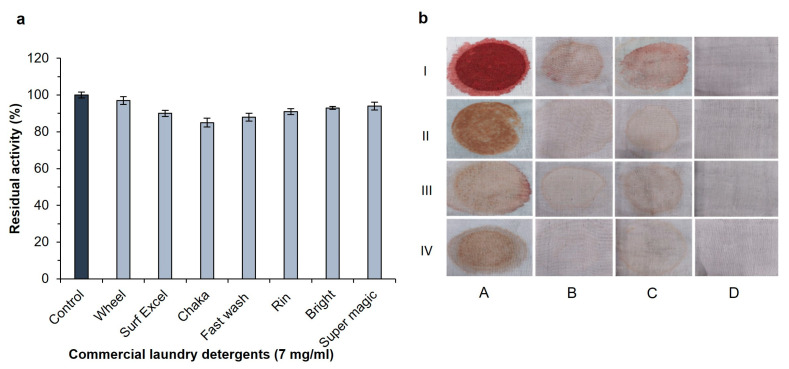
(**a**). Effect of several solid industrial laundry detergents on protease SH21 from *Bacillus siamensis* CSB55. To check the stability of protease in the presence of some industrial solid laundry detergents, the enzyme was incubated with or without detergents at room temperature for 24 h, and residual activity was determined under standard assay conditions. Each value represents the mean (n = 3) ± standard deviation. (**b**) Stain removal ability of SH21. Stained clothes with (I) blood, (II) chocolate, (III) jam, and (IV) tomato sauce, where (A) control (untreated stained clothes pieces or treated with, (B) enzyme alone (200 U/mL), (C) Wheel detergent alone (7 mg/mL), (D) Wheel detergent (7 mg/mL) + enzyme (200 U/mL).

The alkaline proteases are employed in numerous industries, including laundry, dishwashing textiles, and other commercial purposes [40]. The enzymes used in the detergent industry should not drop their enzymatic characteristics in the existence of commercial detergents. Several commercial laundry detergents were pre-incubated with enzymes for 1 h at 30 °C to investigate the stability and compatibility of alkaline proteases with detergents. The enzyme SH21 showed high stability with different commercial detergents (Figure 5a). Protease SH21 retained the highest original activity (97%) with the addition of Wheel, and it maintained 90, 91, 93, and 94% activity when incubated with Surf excel, Rin, Bright, and Super magic, respectively. Moreover, the enzyme showed more than an 85% initial activity when adding Chaka and Fast wash. These attributes undoubtedly revealed that protease SH21 could be used in detergent industries as an additive. In contrast, protease from *B. amyloliquefaciens* showed less than 50% activity after adding the Wheel detergent [41]. 

To evaluate the stain removal ability, several cotton fabrics (5 × 4 cm) were stained with blood, chocolate, jam, and tomato sauce. (Figure 5b). Compared to distilled water (control) and the detergent alone, the enzyme was more effective in removing various stains from cotton fabrics. It was found that treating stained clothes with detergent supplemented with protease gave an excellent result rather than just using detergent on its own. Several reports indicated that due to the lack of protease action in detergents, some stains could not be removed by only protease treatment alone [42,43]. We found that protease SH21 showed very promising results when supplemented with a detergent for washing stained fabric clothes. These findings suggested that SH21 protease may enhance the ability of detergent formulations to remove stains from washed clothing, and it would be a perfect candidate for application in the industrial sectors.

#### 2.5.2. Antimicrobial Activity of Protease SH21

The antimicrobial activity of SH21 was investigated in terms of the minimum inhibitory concentration (MIC) against several gram-positive and gram-negative bacteria. In the present study, we compared the antimicrobial activity of protease SH21 with two reference antibiotics (Bacitracin and Vancomycin) (Table 4). Researchers in the pharmaceutical industry face challenges due to the emergence of multidrug resistance. However, there are attempts to address the current antibiotic crisis, and microbial proteases have gained attention as an optimistic resource to explore new antimicrobial agents [44]. Some gram-positive bacteria, especially *S. aureus,* are known to cause skin diseases, respiratory complications, and food poisoning. Protease SH21 showed a strong antagonistic effect against some pathogenic bacteria similar to other microbial proteases from *Bacillus* sp. that were previously reported to have antimicrobial potential [10,45]. Protease SH21 displayed intense antimicrobial activity against *S. aureus* and *B. subtilis* with an MIC value of 16 µg/mL, which is significantly lower than the two reference antibiotics. The effect of SH21 was better than bacitracin against *E. coli*, *M. luteus*, and *M. smegmatis*. In addition, SH21 showed a similar effect to bacitracin against *P. aeruginosa* and *S. typhimurium*. Gram-negative bacteria showed a higher minimum inhibitory concentration (MIC) than Gram-positive bacteria, indicating that Gram-positive bacteria are very susceptible to our enzyme. The antibacterial properties of SH21 were better in comparison with other previously reported proteases [46,47]. These results indicated that protease SH21 has potent antimicrobial activity.

#### 2.5.3. Antibiofilm Activity of Protease SH21

**Figure 6:**
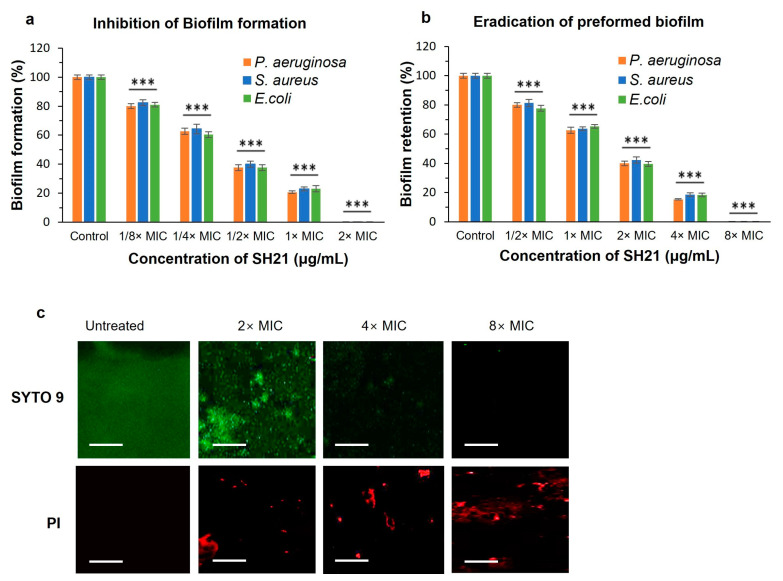
Antibiofilm effect of protease SH21. (**a**) Inhibition of biofilm formation and (**b**) eradication of preformed biofilms against *P. aeruginosa*, *S. aureus*, and *E. coli*. (**c**) Effects of protease SH21 on *P. aeruginosa* 48 h-old biofilms evaluated by applying confocal laser scanning microscopy (CLSM). *P. aeruginosa* was incubated alone or with three different concentrations (2× MIC, 4× MIC, and 8× MIC) of SH21. Live–dead viability staining (SYTO 9/PI) was used to visualize biofilms. Green fluorescence (SYTO 9) and red fluorescence (PI) indicated live and dead cells, respectively. Scale Bars = 50 μm. “***” indicate statistically significant differences at *p* < 0.001.

Biofilm is made up of several microbes implanted in independently created external polymer materials. Due to the extracellular polymeric substances of biofilms, it plays a significant role, acting as a barrier or inhibiting the interaction of antimicrobial agents with bacterial cells. Generally, it needs a higher concentration of antimicrobial agents to inhibit the biofilm than planktonic bacteria cells [48]. In this study, we investigated the antibiofilm activity of SH21 to determine the MBIC and MBEC against three pathogenic bacteria using a crystal violet assay. SH21 exhibited good antibiofilm activity against *P. aeruginosa*, *S. aureus*, and *E. coli*. Biofilm formation rates were around 20% for all examined bacteria at 1× MIC concentration compared to the control (Figure 6a). SH21 inhibited 99.9% of the biofilm formation against *P. aeruginosa* at 2× MIC concentration, and a similar effect was also observed on *S. aureus* and *E. coli* with the same concentration. SH21 exhibited an MBIC of 128 µg/mL against *P. aeruginosa*, and 32 µg/mL for both *S.aureus* and *E. coli* (Table 4).

In another experiment, we found that SH21 could eradicate preformed biofilms (Figure 6b). Removing preformed biofilms is much more difficult than preventing biofilm formation because many intricate elements exist in the solid-designed structures. SH21 eradicated more than 80% (retained less than 20%) of the preformed biofilm at a 4× MIC concentration, and it retained only 0.01% of the biofilm when treated with an 8× MIC concentration against all tested bacteria. The MBEC value of protease SH21 was 512 µg/mL for *P. aeruginosa,* while it was 128 µg/mL against *E. coli* and *S. aureus* (Table 4). In addition, we explored the effect of SH21 on preformed biofilm using confocal microscopy analysis in a dose-dependent manner (Figure 6c). *P. aeruginosa* biofilms were cultured for 48 h and treated with three concentrations of SH21 (2× MIC, 4× MIC, and 8× MIC) followed by incubation for 1 h and stained with green fluorescence SYTO 9 (live cells) and red fluorescence PI (dead cells). As a result, the control group of bacteria was aggregated into a large quantity of biofilms with the highest number of living bacterial cells producing green fluorescence, and it did not display red fluorescence due to the intact living cells. This result suggested that the bacteria incubated in an untreated condition were unable to pass through the PI, and gradually increasing the concentration of SH21 increased the number of dead cells (red) relative to the green-stained living cells of the biofilm. Overall, these observations indicated that SH21 destroys the bacterial biofilm and substantially removes the biofilm cells, implying that it would be a potential therapeutic approach for wound treatment.

## 3. Materials and Methods

### 3.1. Materials

Casein, azocasein, and beef extract were bought from Sigma-Aldrich (St. Louis, MO, USA). Agar powder was procured from Daejung (Siheung-si, Republic of Korea). Peptone and tryptone were obtained from Neogen corp. (Lansing, MI, USA). Sephadex G-75 and Sepharose CL-6B column was purchased from Pharmacia (Uppsala, Sweden). All analytical-quality chemicals and reagents were used in the experiments.

### 3.2. Microorganism and Phylogenic Tree Analysis

Samples of kimchi, a typical traditional Korean fermented vegetable, were collected from various South Korean provinces. For isolating bacteria, 1 g of kimchi was mixed with 0.85% NaCl solution and then incubated at 37 °C for 24 h. For adjusting the colony-forming unit (CFU), the mixed samples were streaked on the Mueller-Hinton agar (MHA) plate composed of 0.2% beef extract, 1.75% acid digest of casein, 0.15% starch, and 0.17% agar powder (*w*/*v*) after being serially diluted up to 10^−7^. Appropriately diluted samples were preserved in glycerol (20%) at −80 °C. Eighty strains were streaked in casein-agar plates containing 1% casein mixed with 0.5% peptone, 0.5% tryptone, 0.01% MgSO_4_.7H_2_O, 0.01% CaCl_2_, 0.03% KH_2_HPO_4_, and 1.5% agar for primary screening, and plates were incubated at 37 °C for 24 h. Congo red (0.5%) was used to swamp the cultured plates for 30 min, and then washed with distilled water. Afterwards, the plates were filled with 1M NaCl solution for 20 min and cleaned with distilled water to detect a clear zone of casein hydrolysis. Primarily selected strains were cultured in a protease production medium at 37 °C for 96 h, and the protease activity was observed at every 4 h interval. The strain CSB55 exhibited the highest protease activity and was chosen for the current study. The identification of this strain was carried out according to Bergey’s Manual of Systemic Bacteriology based on morphologic properties and 16S rRNA gene sequence analysis.

### 3.3. Media and Culture Conditions

Protease production was performed by a *Bacillus siamensis* CSB55 strain in a medium consisting of (g/L): casein, 10; peptone, 5; tryptone, 5; MgSO_4_.7H_2_O, 0.1; KH_2_HPO_4_, 0.2; CaCl2, 0.1; KH_2_HPO_4_, 0.1; pH 8.5. To make the seed culture inoculum, Luria-Bertani (LB) broth medium was used, composed of (g/L): peptone, 10; yeast extract, 5; NaCl, 0.5; pH 7.0, and then it autoclaved at 121 °C for 20 min. A 1% seed culture was added to a 2L Erlenmeyer baffle flask containing 300 mL protease production media and incubated for 64 h at 37 °C with constant shaking at 160 rpm. The cultured medium was centrifuged at 10,000 rpm for 30 min at 4 °C, and the supernatant was utilized as a crude protease. 

### 3.4. Protease Assay 

The activity of the protease enzyme was estimated according to Benmebarek et al. [49] with minor modifications. In short, 150 μL of the substrate solution (azocasein 0.5% dissolved in 20 mM Tris-HCl buffer) was mixed with 350 μL of the enzyme sample. The mixer was incubated at 55 °C for 30 min. Then, 500 μL of TCA (10% *v*/*v*) was added to terminate the reaction and was kept at room temperature for 30 min. Then, the centrifuge was carried out at 10,000 rpm for 10 min to remove undigested protein. Later, 200 μL of supernatant was mixed with 800 μL of 1 N NaOH solution. The absorbance was taken at 440 nm. One unit (U) of protease activity was specified as the amount of enzyme activity that results in a change in the optical density of 0.01 at 440 nm under standard assay conditions. 

### 3.5. Purification of Protease SH21

After *Bacillus siamensis* CSB55 strain was grown for 64 h, the cell-free supernatant was collected by centrifugation at 10,000 rpm for 30 min. The protease-containing supernatant was subjected to the subsequent purification procedures. At first, ammonium sulfate (40–80%) was added to the supernatant by softly stirring and was kept overnight at 4 °C. Then, it was re-centrifuged and precipitation pellets were suspended in a required volume of Tris-HCl buffer (20 mM, pH 9.0). The enzyme sample was subjected to the Sepharose CL-6B column (80 cm × 1.8 cm), which was previously equilibrated with a Tris-HCl buffer (20 mM, pH 9.0). Fractions were collected with an elution rate of 0.30 mL/min. Then, lyophilization was performed to concentrate the active fractions. The concentrated sample was loaded into a Sephadex G-75 column (20 cm × 2.0 cm), and protein elution was performed utilizing the same buffer. After that, active protease fractions were collected, they were lyophilized, and their purity was analyzed. The purified protease was used for other characteristics and applications. 

### 3.6. Protein Measurements, SDS-PAGE, and Zymogram Analysis

The total protein content was estimated according to the Bradford method [50], and the bovine serum albumin (BSA) standard was used. Sodium dodecyl sulfate-polyacrylamide gel electrophoresis (SDS-PAGE) was performed using 12% (*w*/*v*) separating and 5% (*w*/*v*) stacking gel, as reported by Laemmli et al. [51]. Coomassie Brilliant Blue R-250 (Bio-Rad Laboratories, Inc., Hercules, CA, USA) was used to stain the gel to visualize the protein bands. PageRuler Prestained protein ladder (10–170 kDa) was used as a standard protein marker. As described earlier, zymography staining was done by using casein as a substrate [52]. Following electrophoresis, the gel was washed twice for 1 h with 2.5% (*v*/*v*) Triton X-100 to substitute the SDS and separation buffer in the gel. Then the gel was incubated at 40 °C for 1 h in Tris-HCl buffer (pH 9.0) containing 1% (*w*/*v*) casein to produce a cleared area at the site of the proteolytic band. Afterwards, the gel was fixed by using 20% (*w*/*v*) ice-cold TCA for 1 h followed by staining with Coomassie Brilliant Blue R-250 (0.1%, *w*/*v*) in a solution containing water/methanol/acetic acid (50:40:10), and distaining was done with the same solution in the absence of the dye.

### 3.7. The N-terminal Amino Acid Sequencing

To determine the N-terminal amino acid sequence, purified protease SH21 was subjected to the Edman degradation method using a Procise Model 492 protein sequencer (Applied Biosystems, Foster, CA, USA).

### 3.8. Biochemical Characterization of Purified Protease SH21

#### 3.8.1. Determination of Optimum pH and Stability 

To investigate the influence of pH on the enzyme activity, azocasein (0.5%) was used as a substrate, and an assay was performed by a conducting enzyme (0.01 mg) with twelve buffers ranging from pH 2 to 13 at 55 °C for 1 h. The pH stability was also observed by incubating the enzyme with different pH buffer solutions at 40 °C for 24 h. The enzyme activities were calculated under standard assay conditions. The subsequent buffer systems were utilized: 20 mM glycine-HCl for pH 2–3; acetate buffer for pH 4–6; Tris-HCL for pH 7–8; glycine-NaOH for pH 9–11; and KCl-NaOH for pH 12–13.

#### 3.8.2. Determination of Optimum Temperature and Thermal Stability 

To evaluate the effect of temperature on the enzymatic activity, purified protease (0.01 mg) was incubated at numerous temperatures between 30 and 80 °C with an interval of 5 °C for 1 h. The thermostability of the protease was analyzed by incubating the enzyme solution at several temperatures (40, 50, 60, 70, 80, and 90 °C) for 24 h. Proteolytic activity was estimated by applying azocasein as a substrate. The activity of the non-heated sample was taken as 100%.

#### 3.8.3. Effect of Inhibitors, Metals, Surfactants, and Bleaching Agents on Protease Activity 

To check the effect of protease inhibitors on the enzyme activity, purified SH21 (0.01 mg) was incubated with PMSF, DFP, TLCK, TPCK, SBTI, benzamidine, iodoacetamide, 2-mercaptoethanol, leupeptin, pepstatin A, DTNB, EDTA, and EGDA for 1 h at 55 °C. The enzyme activity was employed under standard assay conditions and considered 100% in the absence of inhibitors. 

The effects of a certain number of monovalent and divalent metallic ions (3 mM), surfactants such as SDS (1% *w*/*v*), Tween 20, Tween 80, Triton X-100 (1% *v*/*v*), and bleaching agents (1% *v*/*v*) H_2_O_2_, and NaClO, were investigated by preincubating with 0.01 mg of the protease enzyme at 55 °C for 1 h. The activity of the protease was determined under standard conditions. Without any reagents, the activity was taken as 100%.

#### 3.8.4. Effect of Organic Solvents on Protease Activity

Different concentrations (25%, 50%, and 100% *v*/*v*) of various organic solvents, including DMSO, ethanol, methanol, butanol, acetonitrile, acetone, chloroform, cyclohexane, hexane, decane, and toluene were incubated with the enzyme (0.01 mg) at room temperature for 24 h. The residual activity was calculated under standard assay conditions. The enzyme activity in the absence of any solvent was considered 100%. 

#### 3.8.5. Substrate Specificity and Kinetics Parameters of Protease SH21

To evaluate the hydrolytic activity of protease SH21, the enzyme solution (0.01 mg) was incubated with various substrates (1% *w*/*v*) such as casein, azocasein, skim milk, gelatin, egg albumin, and collagen. The proteolytic activities were assessed under standard assay conditions. In order to analyze the kinetic parameters, the Michaelis constant (K_m_) and the maximum velocity rate (V_max_) were calculated using a Lineweaver–Burk plot. To perform the kinetic test, different azocasein substrate concentrations (0.6–10.0 mg/mL) were applied. 

### 3.9. Application of Purified Protease SH21 

#### 3.9.1. Stability and Compatibility of Protease with Commercial Laundry Detergents

Purified protease was investigated for stability and compatibility with currently commercialized laundry detergents. The detergents utilized in this experiment included Wheel, Surf Excel, Rin (Unilever, Mumbai, India), Chaka (Square Toiletries, Dhaka, Bangladesh), Fast wash (Kohinoor Chemical, Dhaka, Bangladesh), Bright (Mukunghwa, Seoul, Republic of Korea), and Super magic (Green Tech, Gwangju, Republic of Korea). The desired concentration of 7 mg/mL was achieved to make rinsing conditions by diluting the mentioned detergents with tap water. Before adding the enzyme, diluted detergents were boiled at 70 °C for 1 h to deactivate the endogenous protease of laundry detergents. Then, the enzyme was incubated with detergents at 40 °C for 1 h, and the residual activity was examined under standard assay conditions. The enzyme activity with no detergent was taken as 100%. 

#### 3.9.2. Stain Removal Ability of Protease SH21

An experiment was carried out to check the stain removal activity and efficacy of protease SH21, according to Mechri et al., with minor modifications [53]. After staining with blood, chocolate, jam, and tomato sauce, cotton cloth pieces (5 × 4 cm) were air-dried. The stained clothes were washed at 50 °C with a shake incubated at 150 rpm for 1 h using a 50 mL flask containing distilled water alone and with wheel detergents (7 mg/mL), protease SH21 alone (200 U/mL), and wheel detergent + Protease SH21. A visual examination was performed to evaluate the stain removal and efficacy of the enzyme. 

#### 3.9.3. Determination of Minimum Inhibitory Concentration (MIC) of Protease SH21

The antibacterial activity of protease SH21 was evaluated by determining the minimal inhibitory concentration (MIC) against different pathogenic bacteria. The microdilution technique was used to define the MIC value using Mueller Hinton broth (MHB), which was reported before [54]. In short, a two-fold serial dilution of SH21 was added to the microtiter plate and then diluted cultured bacterial suspension (2 × 10^6^ CFU/mL) was added subsequently. The plate was incubated for 24 h at 37 °C. The experiment was done three times for each sample and bacterium. The analyzed microorganisms were *E. coli* KCTC 1923, *P. aeruginosa* KCTC 1637, *S. typhimurium* KCTC 1925, *S. aureus* KCTC 1928, *M. luteus* ATCC 9341, *B. subtilis* ATCC 6633, and *M. smegmatis* ATCC 9341. The MIC was defined as bacterial growth that is entirely inhibited at the lowest concentration of antimicrobial agents with a change in the absorption of less than 0.05.

#### 3.9.4. Inhibition of Biofilm Formation (MBIC Assay)

The effect of SH21 on the biofilm formation was assessed by determining the minimum biofilm inhibitory concentration (MBIC) using crystal violet staining in 96-well plates, as reported earlier [55]. Briefly, 100 μL of the three bacterial cells (*P. aeruginosa*, *S. aureus*, and *E. coli*) were placed in varying concentrations of the enzyme SH21 from 1/8× MIC to 2× MIC and incubated at 37 °C for 48 h. After that, planktonic cells were softly withdrawn, and the plates were washed three times with PBS (10 mM) buffer. To stain the biofilms, a 0.1% crystal violet solution was applied to each well for 30 min. Afterward, deionized water was used twice to rinse each well of the unbound dye and air-dried. For dissolving the remaining crystal violet, 100 μL of ethanol was added. Biofilm biomass was defined as 100% for the control group (without SH21 treatment). The absorbance was measured at 595 nm using a microplate reader. The minimum biofilm inhibition concentration (MBIC) was defined as the lowest antimicrobial concentration that inhibits the growth of biofilms (99.99%), or an increase in 10% of optical density over the initial measurement [56].

#### 3.9.5. Eradication of Preformed Biofilm (MBEC Assay)

The MBEC assay was used to eradicate the performed biofilm, as studied earlier. Microtiter 96-well plates were seeded with suspended bacteria (1 × 10^8^ CFU/mL) and cultured at 37 °C to generate biofilms. A total of 48 h later, planktonic bacteria were separated, leaving deposited biofilms at the bottom side. Then, SH21 at different concentrations (from 1/2× MIC to 8× MIC) was added to treat these preformed biofilms for 12 h. After that, the supernatant was removed carefully and stained the remaining biofilms with an addition of 0.1% crystal violet. The following steps were the same as above. The MBEC was defined as the lowest antimicrobial agent concentration that eradicates 99.9% of preformed biofilm cells (3 log10 reductions in CFU/mL) [57]. All experiments were performed three times.

#### 3.9.6. Confocal Laser Scanning Microscopy Analysis

A multidrug resistance pathogenic bacterium, *P. aeruginosa,* were cultured in 24-well plates in MHB glucose medium containing glass coverslips to form biofilms for 48 h. After the biofilms were grown on glass coverslips, planktonic bacteria were removed, and the biofilms were washed with a PBS buffer three times. Then, glass coverslips were transferred to fresh 24-well plates containing three different concentrations (2× MIC, 4× MIC, and 8× MIC) of SH21 and incubated for 6 h. After incubation, coverslips were washed two times with a PBS buffer, and the LIVE/DEAD BacLight Bacterial kit (6.7 µM SYTO 9 and 40 µM Propidium iodide) was used to stain the cells. After incubation in a dark place at 37 °C for 20–30 min, images were taken using (63X) CLSM (Carl Zeiss LSM510 microscope, Jena, Germany).

### 3.10. Statistical Analysis

All experiments were carried out three times and the results were presented as mean (±) standard deviation. Student’s t-test or one-way analysis of variance (ANOVA) were used to determine the statistical significance using IBM SPSS statistics V22.0. Statistically significant results were defined as *p* *** < 0.001, *p* **< 0.01, and *p* * < 0.05. 

## 4. Conclusions

In the current study, we found a novel alkaline protease SH21 produced by the *Bacillus siamensis* CSB55 strain with some prominent characteristics. Purified protease showed homogeneity on the SDS-PAGE, and the molecular weight was 25 kDa. The results demonstrated that it was highly stable and effective at a broad scale of pH and temperature with the highest of 9.0 and 55 °C. It also displayed excellent tolerability and stability even upon exposure to several organic solvents, surfactants, and commercial laundry detergents. Considering these characteristics, it may be used in laundry detergent formulation as an additive. In addition, SH21 showed good antibacterial and antibiofilm activity against some pathogenic bacteria. Based on these findings, SH21 may serve as a promising candidate for bacterial infections and antibiofilm treatments. Future research is needed to understand how protease SH21 functions against pathogenic bacteria and how it can be used in other bio-industrial applications.

## Figures and Tables

**Table 1:** Purification steps of protease SH21 from *Bacillus siamensis* CSB55.

Purification Steps	Total Protein (mg)	Total Activity (U)	Specific Activity (U/mg)	Purification Fold	Recovery (%)
Cell-free supernatant	256	32,454	126.77	1.00	100
Ammonium sulphate	132	24,475	185.42	1.46	75.41
Sepharose CL-6B	24	16,562	690.08	5.44	51.03
Sephadex G-75	1.8	5268	2926.67	23.09	16.23

**Table 2:** Alignment of the N-terminal acid sequence of purified protease SH21 with theoretical proteins from various *Bacillus* sp.

SN	Microorganisms	N-terminal Amino Acid Sequences	Identity (%)	NCBI References
1	*Bacillus siamensis*	QTGGSFFEPFNSYNSGLWQKANGYS	100	Current study
2	*Bacillus halotolerans*	QTGGSFFDPFNSYNSGLWQKANGYS	96	WP_059335710.1
3	*Paenibacillus macerans*	QTGGSFFEPFNSYNSGTWEKADGYS	88	1U0A_A
4	*Bacillus licheniformis*	QTGGSFYEPFNNYNTGLWQKADGYS	84	1GBG_A
5	*Bacillus subtilis*	QTGGSFFDPFNGYNSGFWQKADGYS	84	3O5S_A
6	*Bacillus subtilis*	GSVFWEP-KSYFNPSTWEKADGYS	58.33	1AXK_A

**Table 3:** Effects of various inhibitors, metal ions, surfactants, and bleaching agents on purified protease SH21.

Inhibitors, Metals, Surfactants and Bleaching Agents	Concentrations	Residual Activity (%)
None	-	100 ± 2.2
PMSF	5 mM	10 ± 1.8
DFP	5 mM	12 ± 1.5
TLCK	1 mM	102 ± 1.2
TPCK	1 mM	101 ± 1.5
SBTI	3 mg/mL	103 ± 1.7
Benzamidine	5 mM	104 ± 1.3
Iodoacetamide	5 mM	95 ± 2.3
2-mercaptoethanol	5 mM	90 ± 1.8
Leupeptin	50 μg/mL	96 ± 1.9
Pepstatin A	10 μg/mL	93 ± 1.4
DTNB	10 mM	92 ± 1.6
EDTA	10 mM	87 ± 1.8
EGTA	1 mM	85 ± 1.4
Mg^2+^	3 mM	135 ± 1.3
Zn^2+^	3 mM	125 ± 1.7
Mn^2+^	3 mM	120 ± 2.4
Fe^2+^	3 mM	145 ± 2.0
Na^+^, Ca^2+^, Cu^2+^	3 mM	100 ± 1.5
Ni^2+^, Co^2+^, Cd^2+^, Hg^2+^	3 mM	10 ± 1.2
SDS	1% (*w*/*v*)	85 ± 1.3
Tween 20	1% (*v*/*v*)	90 ± 1.6
Tween 80	1% (*v*/*v*)	88 ± 1.8
Triton X-100	1% (*v*/*v*)	92 ± 1.5
H_2_O_2_	1% (*v*/*v*)	80 ± 1.2
NaClO	1% (*v*/*v*)	83 ± 1.6

**Table 4:** Antibacterial (MIC) and antibiofilm (MBIC and MBEC) activity of protease SH21.

Microorganisms	MIC (µg/mL)			MBIC (µg/mL)	MBEC (µg/mL)
	Protease SH21	Bacitracin	Vancomycin	Protease SH21	Protease SH21
**Gram-negative bacteria**					
*Escherichia coli* KCTC 1923	16	128	128	32 (2× MIC)	128 (8× MIC)
*Pseudomonas aeruginosa* KCTC 1637	64	64	2	128 (2× MIC)	512 (8× MIC)
*Salmonella typhimurium* KCTC 1925	32	32	2		
**Gram-positive bacteria**					
*Staphylococcus aureus* KCTC 1928	16	128	64	32 (2× MIC)	128 (8× MIC)
*Micrococcus luteus* ATCC 9341	32	128	128		
*Bacillus subtilis* ATCC 6633	16	64	32		
*Mycobacterium smegmatis* ATCC 9341	16	32	2		

MIC, minimum inhibitory concentration. MBIC, minimum biofilm inhibitory concentration. MBEC, minimum biofilm eradication concentration.

## References

[1] Gupta R., Beg Q., Lorenz P. (2002). Bacterial alkaline proteases: Molecular approaches and industrial applications. Appl. Microbiol. Biotechnol..

[2] Kumar C.G., Takagi H. (1999). Microbial alkaline proteases: From a bioindustrial viewpoint. Biotechnol. Adv..

[3] Mei C., Jiang X. (2005). A novel surfactant-and oxidation-stable alkaline protease from Vibrio metschnikovii DL 33–51. Process Biochem..

[4] Samal B.B., Karan B., Stabinsky Y. (1990). Stability of two novel serine proteinases in commercial laundry detergent formulations. Biotechnol. Bioeng..

[5] Bhaskar N., Sudeepa E., Rashmi H., Selvi A.T. (2007). Partial purification and characterization of protease of Bacillus proteolyticus CFR3001 isolated from fish processing waste and its antibacterial activities. Bioresour. Technol..

[6] Patel R.K., Dodia M.S., Joshi R.H., Singh S.P. (2006). Purification and characterization of alkaline protease from a newly isolated haloalkaliphilic *Bacillus* sp. Process Biochem..

[7] Jacobs M., Eliasson M., Uhlén M., Flock J.-I. (1985). Cloning, sequencing and expression of subtilisin Carlsberg from Bacillus licheniformis. Nucleic Acids Res..

[8] Wells J.A., Ferrari E., Henner D.J., Estell D.A., Chen E.Y. (1983). Cloning, sequencing, and secretion of Bacillus amyloliquefaciens subtillisin in Bacillus subtilis. Nucleic Acids Res..

[9] de Kraker M.E., Stewardson A.J., Harbarth S. (2016). Will 10 million people die a year due to antimicrobial resistance by 2050?. PLoS Med..

[10] Rachanamol R., Lipton A., Thankamani V., Sarika A., Selvin J. (2017). Production of protease showing antibacterial activity by Bacillus subtilis VCDA associated with tropical marine sponge Callyspongia diffusa. J. Microb. Biochem. Technol.

[11] Siritapetawee J., Thammasirirak S., Samosornsuk W. (2012). Antimicrobial activity of a 48-kDa protease (AMP48) from Artocarpus heterophyllus latex. Eur. Rev. Med. Pharmacol. Sci..

[12] Thomas N.N., Archana V., Shibina S., Edwin B.T. (2021). Isolation and characterization of a protease from *Bacillus* sps. Mater. Today: Proc..

[13] Donlan R.M. (2002). Biofilms: Microbial life on surfaces. Emerg. Infect. Dis..

[14] Worthington R.J., Blackledge M.S., Melander C. (2013). Small-molecule inhibition of bacterial two-component systems to combat antibiotic resistance and virulence. Future Med. Chem..

[15] Teitzel G.M., Parsek M.R. (2003). Heavy metal resistance of biofilm and planktonic Pseudomonas aeruginosa. Appl. Environ. Microbiol..

[16] Algburi A., Comito N., Kashtanov D., Dicks L.M., Chikindas M.L. (2017). Control of biofilm formation: Antibiotics and beyond. Appl. Environ. Microbiol..

[17] Fleming D., Rumbaugh K.P. (2017). Approaches to dispersing medical biofilms. Microorganisms.

[18] Lister J.L., Horswill A.R. (2014). Staphylococcus aureus biofilms: Recent developments in biofilm dispersal. Front. Cell. Infect. Microbiol..

[19] Karray A., Alonazi M., Horchani H., Ben Bacha A. (2021). A novel thermostable and alkaline protease produced from Bacillus stearothermophilus isolated from olive oil mill sols suitable to industrial biotechnology. Molecules.

[20] Guleria S., Walia A., Chauhan A., Shirkot C.K. (2016). Purification and characterization of detergent stable alkaline protease from Bacillus amyloliquefaciens SP1 isolated from apple rhizosphere. J. Basic Microbiol..

[21] Kim W., Kim S. (2005). Purification and characterization of Bacillus subtilis JM-3 protease from anchovy sauce. J. Food Biochem..

[22] Öztürk S., Özeren-Morgan M., Dilgimen A.S., Denizci A.A., Arikan B., Kazan D. (2009). Alkaline serine protease from halotolerantBacillus licheniformis BA17. Ann. Microbiol..

[23] Lee S., Lee J., Jin Y.-I., Jeong J.-C., Chang Y.H., Lee Y., Jeong Y., Kim M. (2017). Probiotic characteristics of Bacillus strains isolated from Korean traditional soy sauce. LWT-Food Sci. Technol..

[24] Van Kampen V., Merget R. (2002). Occupational airway sensitization due to subtilisin. Pneumol. (Stuttg. Ger.).

[25] Gupta R., Gupta K., Saxena R., Khan S. (1999). Bleach-stable, alkaline protease from *Bacillus* sp. Biotechnol. Lett..

[26] Özçelik B., Aytar P., Gedikli S., Yardımcı E., Çalışkan F., Çabuk A. (2014). Production of an alkaline protease using Bacillus pumilus D3 without inactivation by SDS, its characterization and purification. J. Enzym. Inhib. Med. Chem..

[27] Silva C.R.d., Delatorre A.B., Martins M.L.L. (2007). Effect of the culture conditions on the production of an extracellular protease by thermophilic *Bacillus* sp. and some properties of the enzymatic activity. Braz. J. Microbiol..

[28] Sana B. (2015). Marine microbial enzymes: Current status and future prospects. Springer Handbook of Marine Biotechnology.

[29] Rao M.B., Tanksale A.M., Ghatge M.S., Deshpande V.V. (1998). Molecular and biotechnological aspects of microbial proteases. Microbiol. Mol. Biol. Rev..

[30] Park J.W., Park J.E., Choi H.K., Jung T.W., Yoon S.M., Lee J.S. (2013). Purification and characterization of three thermostable alkaline fibrinolytic serine proteases from the polychaete Cirriformia tentaculata. Process Biochem..

[31] Hedstrom L. (2002). Serine protease mechanism and specificity. Chem. Rev..

[32] Donaghy J., McKay A. (1993). Production and properties of an alkaline protease by Aureobasidium pullulans. J. Appl. Bacteriol..

[33] Vallee B.L., Ulmer D.D. (1972). Biochemical effects of mercury, cadmium, and lead. Annu. Rev. Biochem..

[34] Haddar A., Bougatef A., Agrebi R., Sellami-Kamoun A., Nasri M. (2009). A novel surfactant-stable alkaline serine-protease from a newly isolated Bacillus mojavensis A21. Purification and characterization. Process Biochem..

[35] Annamalai N., Rajeswari M.V., Sahu S.K., Balasubramanian T. (2014). Purification and characterization of solvent stable, alkaline protease from Bacillus firmus CAS 7 by microbial conversion of marine wastes and molecular mechanism underlying solvent stability. Process Biochem..

[36] Lakshmi B., Kumar D.M., Hemalatha K. (2018). Purification and characterization of alkaline protease with novel properties from Bacillus cereus strain S8. J. Genet. Eng. Biotechnol..

[37] Emon T.H., Hakim A., Chakraborthy D., Bhuyan F.R., Iqbal A., Hasan M., Aunkor T.H., Azad A.K. (2020). Kinetics, detergent compatibility and feather-degrading capability of alkaline protease from Bacillus subtilis AKAL7 and Exiguobacterium indicum AKAL11 produced with fermentation of organic municipal solid wastes. J. Environ. Sci. Health Part A.

[38] Priya J., Divakar K., Prabha M.S., Selvam G.P., Gautam P. (2014). Isolation, purification and characterisation of an organic solvent-tolerant Ca2+-dependent protease from Bacillus megaterium AU02. Appl. Biochem. Biotechnol..

[39] Aguilar J.G.d.S., de Castro R.J., Sato H.H. (2019). Alkaline protease production by Bacillus licheniformis LBA 46 in a bench reactor: Effect of temperature and agitation. Braz. J. Chem. Eng..

[40] Ferrareze P.A.G., Correa A.P.F., Brandelli A. (2016). Purification and characterization of a keratinolytic protease produced by probiotic Bacillus subtilis. Biocatal. Agric. Biotechnol..

[41] Mushtaq H., Jehangir A., Ganai S.A., Farooq S., Ganai B.A., Nazir R. (2021). Biochemical characterization and functional analysis of heat stable high potential protease of Bacillus amyloliquefaciens strain HM48 from soils of Dachigam National Park in Kashmir Himalaya. Biomolecules.

[42] Chen X., Zhou C., Xue Y., Shi J., Ma Y. (2018). Cloning, expression, and characterization of an alkaline protease, AprV, from Vibrio sp. DA1-1. Bioprocess Biosyst. Eng..

[43] Oztas Gulmus E., Gormez A. (2020). Characterization and biotechnological application of protease from thermophilic Thermomonas haemolytica. Arch. Microbiol..

[44] Barzkar N. (2020). Marine microbial alkaline protease: An efficient and essential tool for various industrial applications. Int. J. Biol. Macromol..

[45] Rai S.K., Mukherjee A.K. (2009). Ecological significance and some biotechnological application of an organic solvent stable alkaline serine protease from Bacillus subtilis strain DM-04. Bioresour. Technol..

[46] Abd-ElKhalek A.M., Seoudi D.M., Ibrahim O.A., Abd-Rabou N.S., Abd ElAzeem E.M. (2020). Extraction, partial purification, characteristics, and antimicrobial activity of plant protease from Moringa Oleifera leaves. J. Appl. Biotechnol. Rep..

[47] Muthu S., Gopal V.B., Soundararajan S., Nattarayan K., Narayan K.S., Lakshmikanthan M., Malairaj S., Perumal P. (2017). Antibacterial serine protease from Wrightia tinctoria: Purification and characterization. Plant Physiol. Biochem..

[48] De La Fuente-Núñez C., Cardoso M.H., de Souza Cândido E., Franco O.L., Hancock R.E. (2016). Synthetic antibiofilm peptides. Biochim. Et Biophys. Acta (BBA)-Biomembr..

[49] Benmebarek H., Escuder-Rodríguez J.-J., González-Siso M.-I., Karroub K. (2020). Test for the production and assay of the proteolytic activities of halophilic bacteria and archaea isolated from Algerian hypersaline environments. Multidiscip. Digit. Publ. Inst. Proc..

[50] Bradford M.M. (1976). A rapid and sensitive method for the quantitation of microgram quantities of protein utilizing the principle of protein-dye binding. Anal. Biochem..

[51] Laemmli U.K. (1970). Cleavage of structural proteins during the assembly of the head of bacteriophage T4. Nature.

[52] Jaouadi B., Ellouz-Chaabouni S., Rhimi M., Bejar S. (2008). Biochemical and molecular characterization of a detergent-stable serine alkaline protease from Bacillus pumilus CBS with high catalytic efficiency. Biochimie.

[53] Mechri S., Berrouina M.B.E., Benmrad M.O., Jaouadi N.Z., Rekik H., Moujehed E., Chebbi A., Sayadi S., Chamkha M., Bejar S. (2017). Characterization of a novel protease from Aeribacillus pallidus strain VP3 with potential biotechnological interest. Int. J. Biol. Macromol..

[54] Li F., Weir M.D., Fouad A.F., Xu H.H. (2013). Time-kill behaviour against eight bacterial species and cytotoxicity of antibacterial monomers. J. Dent..

[55] Zeng P., Yi L., Xu J., Gao W., Xu C., Chen S., Chan K.-F., Wong K.-Y. (2021). Investigation of antibiofilm activity, antibacterial activity, and mechanistic studies of an amphiphilic peptide against Acinetobacter baumannii. Biochim. Et Biophys. Acta (BBA)-Biomembr..

[56] Bessa L.J., Eaton P., Dematei A., Plácido A., Vale N., Gomes P., Delerue-Matos C., SA Leite J.R., Gameiro P. (2018). Synergistic and antibiofilm properties of ocellatin peptides against multidrug-resistant Pseudomonas aeruginosa. Future Microbiol..

[57] Thieme L., Hartung A., Tramm K., Klinger-Strobel M., Jandt K.D., Makarewicz O., Pletz M.W. (2019). MBEC versus MBIC: The lack of differentiation between biofilm reducing and inhibitory effects as a current problem in biofilm methodology. Biol. Proced. Online.

